# HIV-driven virome dysbiosis unveils distinct virome features and inter-viral correlations in blood and respiratory niches

**DOI:** 10.1038/s42003-026-10221-z

**Published:** 2026-05-08

**Authors:** Wang Li, Ping Ni, Juan Xu, Xingyue Zhao, Anhua Dou, Yanhuan Wang, Linjie Peng, Shiyin Huang, Yue Chen, Qi Shi, Youhua Xie, Wen Zhang, Shaokun Pan, Chenglin Zhou

**Affiliations:** 1 Clinical Laboratory Center, The Affiliated Taizhou People’s Hospital of Nanjing Medical University, Taizhou, China; 2 Department of Laboratory Medicine, School of Medicine, Jiangsu University, Zhenjiang, China; 3 Shanghai Institute of Infectious Diseases and Biosecurity, Key Laboratory of Medical Molecular Virology (MOE/MHC/CAMS), Shanghai Frontiers Science Center of Pathogenic Microbes and Infection, Department of Microbiology and Parasitology, School of Basic Medical Sciences, Shanghai Medical College, Fudan University, Shanghai, China; 4 Clinical Medical College of Anhui Medical University, Hefei, China

**Keywords:** Metagenomics, Clinical microbiology

## Abstract

While systemic immune dysregulation is well-documented in HIV infection, its impact on blood and respiratory tract viromes remains poorly understood. This study characterizes HIV-associated alterations in viral communities and examines their clinical relevance. Using viral metagenomics, we compare 203 ART-treated HIV-positive individuals and 120 healthy controls. HIV infection significantly restructures the blood virome, shifting from bacteriophage dominance (96.2% in controls) to eukaryotic virus predominance (69.1%). Increased alpha diversity, significant β-diversity divergence, and heightened dispersion heterogeneity are observed in HIV cases. Consistent enrichment of *Flaviviridae*, *Parvoviridae*, and *Anelloviridae* is detected. Throat viromes maintain phage dominance (>90%) but exhibit strain-level diversification, including *Microviridae* proliferation. Network analysis reveals *Retroviridae-Anelloviridae* co-dynamics (r = +0.562) and identifies *Picobirnaviridae* as a key interactor. Functional analysis shows enriched viral replication and host modulation genes. Compartment-specific disruption patterns nominate Pegivirus C, parvovirus B19, and Anelloviruses as potential biomarkers. Cross-kingdom viral interactions suggest novel mechanisms influencing disease progression and support future virome-targeting adjunct therapies.

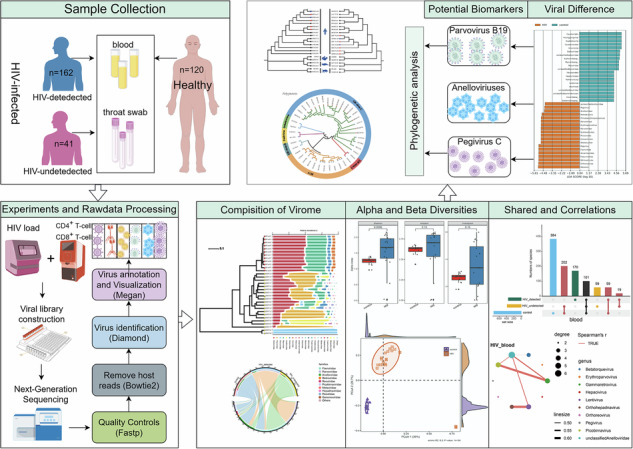

## Abstract (web summary)

HIV infection shifts blood virome from phage to eukaryotic virus dominance, enriches Flaviviridae/Parvoviridae/Anelloviridae, and diversifies throat virome strains, revealing potential viral biomarkers and cross-kingdom interactions.

## Introduction

Human immunodeficiency virus (HIV), a single-stranded RNA retrovirus, causes acquired immunodeficiency syndrome (AIDS) through progressive depletion of CD4^+^ T lymphocytes and systemic immune dysfunction^1–3^. Despite advances in antiretroviral therapy (ART) that have transformed HIV into a chronic condition, critical challenges persist. Residual viral reservoirs, incomplete immune recovery, and chronic inflammation continue to drive heightened susceptibility to opportunistic infections, malignancies, and noncommunicable diseases among people living with HIV/AIDS (PLWHA), a population exceeding 39 million globally as of 2023^4,5^. These unresolved issues necessitate novel strategies to unravel the complex triad of HIV pathogenesis, host immunity, and microbial ecology.

The human microbiome, comprising dynamic communities of bacteria, fungi, and viruses, plays a pivotal role in immune regulation and disease pathogenesis^6^. While metagenomic sequencing has enabled unbiased characterization of microbial communities and revealed HIV-associated disruptions in gut bacterial diversity linked to systemic inflammation^7–10^, research remains disproportionately focused on bacterial components of the microbiome^11,12^. Although a limited number of studies have reported virome profiles in people with HIV^13–15^, the available research remains scarce and insufficient, and the mechanisms by which HIV influences viral community composition across different anatomical sites still require in‑depth exploration.

Viromes, consisting of eukaryotic and prokaryotic viruses, serve as key regulators of microbial homeostasis and immune function^16^. In HIV infection, prolonged immunosuppression may destabilize these communities, enabling opportunistic pathogen expansion while depleting commensal viral populations^17^. Emerging evidence implicates viral co-infections (e.g., cytomegalovirus, Epstein–Barr virus, and torque teno virus) in accelerating HIV progression and end-organ damage^18,19^.

Blood represents a systemic compartment that directly mirrors immune status and circulating viral dynamics, making it a logical target for studying HIV-driven virome alterations. In parallel, the upper respiratory tract, though not a primary reservoir for HIV, serves as a critical immune-mucosal interface that is continuously exposed to environmental and commensal viruses. Oropharyngeal microbes can migrate and colonize the lungs, establishing a direct ecological association with pulmonary health^20^. In PLWHA, persistent immune dysfunction and heightened susceptibility to respiratory infections underscore the clinical relevance of this site^21^. Moreover, studies have shown that the abundance of viruses in the upper respiratory tract, especially certain bacteriophages, appears to be a sensitive indicator of the host’s immune competence^22^. To our knowledge, the upper respiratory tract virome in people living with HIV remains understudied. Therefore, profiling the upper respiratory virome in HIV-infected individuals is essential not only to understand localized viral ecology but also to uncover how mucosal viral communities may signal or perpetuate systemic immune dysregulation.

As blood and the oropharyngeal tract represent critical immune interfaces, their viromes likely reflect both local and systemic host-virus interactions, yet their roles in HIV pathogenesis remain poorly characterized^23^. Investigating the virome of both blood and the upper respiratory tract in HIV-infected patients is essential for elucidating HIV pathogenesis, informing therapeutic strategies, and ultimately improving patient outcomes.

To further investigate these questions, this study conducted a parallel virome analysis of blood and oropharyngeal samples from ART-treated HIV patients and matched controls. We pursue three objectives: (1) taxonomic characterization: mapping HIV-associated alterations in viral diversity and community structure. (2) Biomarker identification: detecting differentially abundant viral taxa with diagnostic or prognostic potential. (3) Mechanistic exploration: analyzing virome-immune system interactions during ART-mediated immune reconstitution. By integrating viral metagenomics with clinical parameters (viral load, CD4^+^/CD8^+^ T-cell counts), this work provides new insights into HIV-associated virome dysregulation and its implications for personalized comorbidity management.

## Results

### Clinical data analysis

**Fig. 1:**
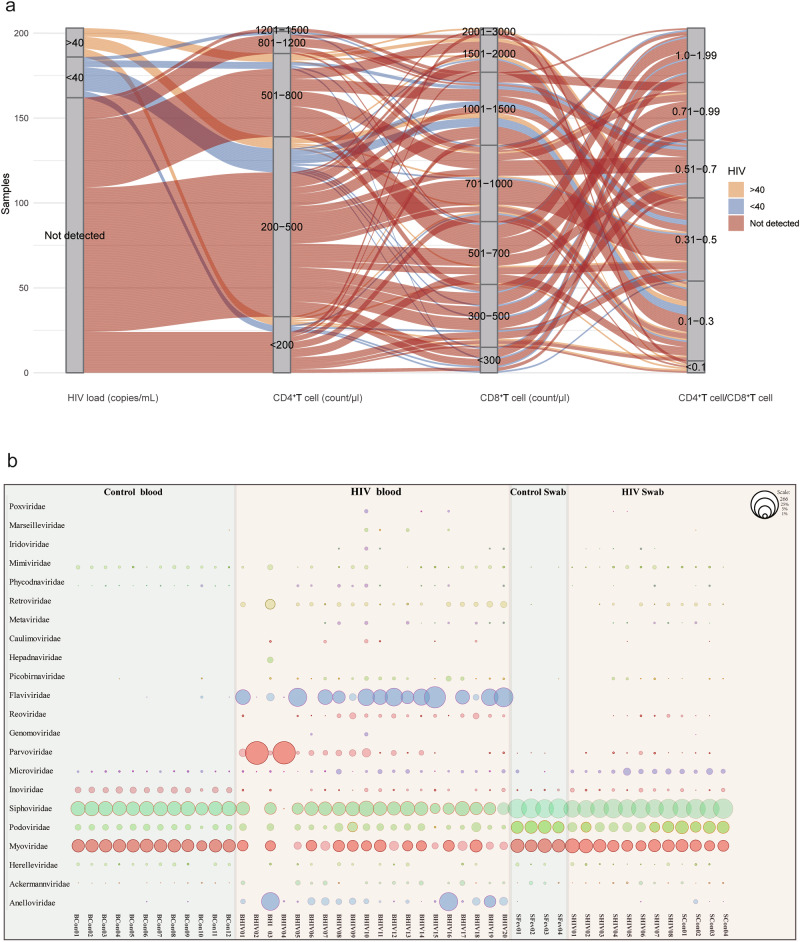
Immune cell distribution and viral family abundance in HIV-infected individuals. **a** The Sankey diagram illustrates the distribution of CD4^+^ T cell and CD8^+^ T cell counts (in cells/μL), and CD4^+^/CD8^+^ ratios in different groups of HIV load (in copies/mL). **b** Overlook the top 22 relative abundance of various viral families in control blood, HIV-infected blood, control swabs, and HIV-infected swabs. The size of each circle represents the relative abundance of a specific viral family within the sample.

Clinical data of participants comprising HIV-infected patients with detectable viremia (HIV-detected), HIV-infected patients with undetectable viremia (HIV-undetected), and healthy controls showed no significant differences in age or gender distribution among the three groups. Viral load quantification revealed 79.8% (162/203) of patients achieved complete HIV RNA suppression (undetectable), while 20.2% (41/203) maintained detectable viremia. Notably, 4.4% (9/203) exhibited high-level replication (>1000 copies/mL), suggesting incomplete therapeutic control. Two samples in the HIV_detected group were CMV-positive, and one sample was EBV-positive. In contrast, the HIV_undetected group had two CMV-positive samples and two EBV-positive samples. Immunological stratification exposed divergent patterns: the HIV-detected group harbored higher rates of severe immunosuppression (CD4^+^ T-cell <200 cells/μl: 63.4% vs. 41.4% in HIV-undetected; *χ*² = 4.76, *p* = 0.04) (Table 1). Viremic patients exhibited higher CD8^+^ T-cell counts and demonstrated significant reductions in both CD4^+^T-cell counts and CD4^+^ T cell/CD8^+^ T cell ratios compared to aviremic counterparts (Supplementary Fig. 1). Detailed results regarding HIV viral load, CMV viral load, EBV viral load, immune cell counts, and other parameters are presented in Fig. 1a, Supplementary Data 1 and 2.

**Table 1:** Sample informations

		HIV_detected	HIV_undetected	Control	*P*
Gender	Male	34	130	94	>0.5*
	Female	7	32	26	
Age (year)		39.5 ± 9.1 (18–55)	40.1 ± 5.2 (20–53)	38.8 ± 7.9 (18–51)	>0.1*
HIV loading (copies/mL)	>40	23	/	/	/
	<40	18	/	/	/
	Not detected	/	162	/	/
CMV	Positive	2	2	/	/
EBV	Positive	1	2	/	/
CD4^+^ T cell (count/μL)	<200 (32–189)	11	22	/	/
	≥200 (205–1305)	30	140	/	/
ATR duration (year)		3.38 ± 0.26 (1.08–8.92)	3.43 ± 0.147 (1.0–12.08)	/	/
CD8^+^ T cell (count/μL) (mean)		852 (124–2474)	678 (34–2905)	/	/
CD4^+^ T cell/CD8^+^ T cell (mean)		0.38 (0.07–1.56)	0.5 (0.04–1.95)	/	/

>0.5✱: no statistically significant differences in gender between the following groups: HIV (HIV_detected + HIV_undetected) vs. control (*p* = 0.595, OR = 1.163 [95% CI: 0.666, 2.031]), HIV_detected vs. HIV_undetected (*p* = 0.697, OR = 1.196 [95% CI: 0.486, 2.942]), HIV_detected vs. control (*p* = 0.529, OR = 1.343 [95% CI: 0.534, 3.378]), and HIV_undetected vs. control (*p* = 0.694, OR = 1.123 [95% CI: 0.628, 2.01]) using Pearson’s chi-squared test.

>0.1✱: no statistically significant differences in age between the following groups: HIV (HIV_detected + HIV_undetected) vs. control (*p* = 0.133, *r* = 0.0836 [95% CI: 0.047, 0.196]), HIV_detected vs. HIV_undetected (*p* = 0.935, Cohen’s *d* = 0.0198 [95% CI: −2.846, 3.089]) and HIV_detected vs. control (*p* = 0.508, Cohen’s *d* = −0.136 [95% CI: −4.156, 2.082]), using Welch’s t-test; HIV_undetected vs. control (*p* = 0.129, Cohen’s *d* = 0.192 [95% CI: −0.341, 2.658]), using Mann–Whitney *U* test.

### Viral population composition

To investigate anatomical variations in virome composition, we analyzed 48 metagenomic libraries across four clinically defined cohorts: HIV-infected blood (*n* = 20; BHIV01–BHIV20), healthy control blood (*n* = 12; BCon01–BCon12), HIV-infected throat swabs (*n* = 12; SHIV01–SHIV12), and healthy throat specimens (*n* = 4; SCon01–SCon04). Participants with HIV were further stratified into viremic (HIV-detected) and aviremic (HIV-undetected) subgroups based on viral load. Furthermore, within the aviremic subgroup, participants were stratified into three categories based on CD4^+^ T-cell count: <200 (named low-CD4), 200–500 (medium-CD4), and >500 (high-CD4) cells/μL. The <200 cells/μL stratum was not analyzed separately owing to insufficient pool size. This stratification enabled a more precise evaluation of the relationship between HIV infection and virome alterations. High-throughput sequencing yielded 109,792 virus-matched reads from blood samples (229–21,254/library) and 225,066 virus-matched reads from throat swab samples (978–99,170/library). Taxonomic classification against the NCBI nr database, identifying 52 viral families in blood versus 43 in throat specimens. Bubble plot visualization (Fig. 1b) revealed two critical patterns: Caudovirales bacteriophages (*Siphoviridae*, *Podoviridae*, and *Myoviridae*) maintained pan-microbiome dominance across all groups, while blood viromes from HIV-infected individuals showed marked enrichment of eukaryotic viral families, particularly *Flaviviridae, Anelloviri*dae, and *Parvoviridae*. This restructuring was most pronounced in viremic patients, suggesting active HIV replication drives specific viral community shifts. The throat virome exhibited remarkable conservation between HIV and control groups, with phage populations maintaining >90% dominance regardless of HIV status, though subtle eukaryotic virus variations emerged at finer taxonomic resolutions.

**Fig. 2:**
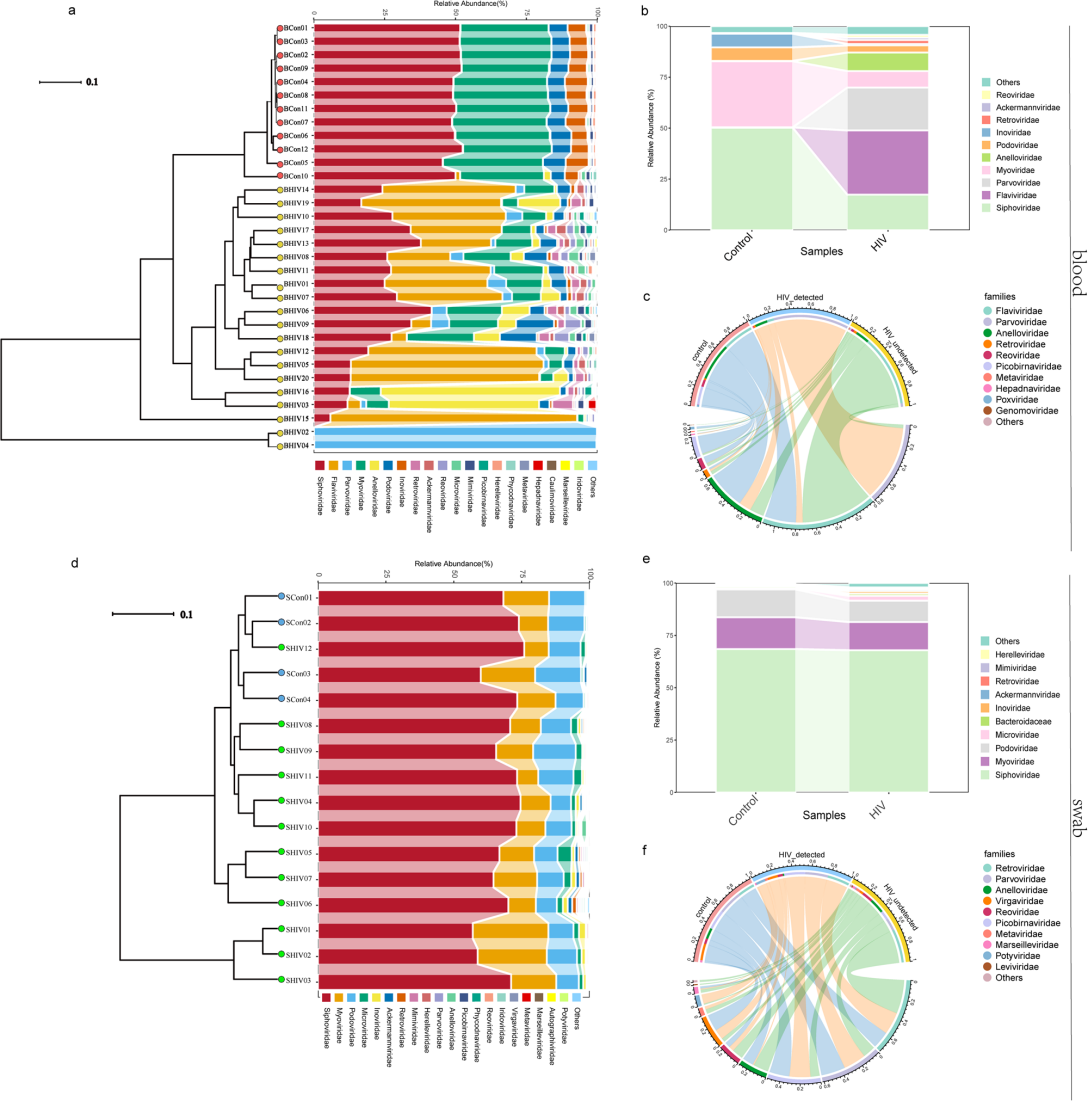
Composition of viral species in blood and throat swab samples. **a** The UPGMA tree in the left panel shows the similarities and evolutionary relationships among distinct viral communities across various samples. The control group is marked with a red circle, and HIV-infected individuals are marked with a blue circle. The panel on the right displays the relative abundance of the top 20 viral families in each sample library. **b** The stacked bar chart shows the relative abundance of the top 10 viral families in blood samples from the healthy control group and the HIV-infected group. **c** The circular plot shows the relative abundance of the top 10 viral families among eukaryotic viruses in blood samples from the healthy control group and the HIV-infected group. The length of the arcs on the circle corresponds to the proportion of each viral family, and different colors represent different viral families. **d** The UPGMA tree and the relative proportions of the top 20 viral families in each sample library are shown for throat swab samples. The control group is marked with a blue circle, and HIV-infected individuals are marked with a green circle. **e** The proportion of the top 10 viral families in throat swab samples from the healthy control group and the HIV-infected group is depicted. **f** The relative abundance of the top 10 viral families among eukaryotic viruses in throat swab samples from the healthy control group and the HIV-infected group is illustrated.

UPGMA clustering revealed fundamental patterns in viral community architecture across cohorts (Fig. 2a, left panel). Control blood samples formed a distinct phylogenetic clade (BCon01–BCon12), while HIV-positive blood specimens clustered separately except for two outliers (BHIV02 and BHIV04). Mirroring these phylogenetic relationships, family-level abundance profiles showed that bacteriophages emerged as the overwhelmingly predominant taxa in the controls, versus marked eukaryotic virus expansion in HIV-infected individuals. Among HIV-infected cohorts, notable heterogeneity was observed. Thirteen libraries showed *Flaviviridae* predominance (18.4–84.0%), BHIV02 and BHIV04 were exclusively dominated by *Parvovirida*e (100%), and BHIV06 and BHIV13 exhibited significant *Anelloviridae* enrichment (>50%) (Fig. 2a right panel). Overall comparison between the two groups revealed that bacteriophages (*Siphoviridae* 50.2%, *Podoviridae* 32.4%, and *Myoviridae* 6.7%) constituting 89.3% of control blood viromes versus marked eukaryotic virus expansion in HIV-infected individuals (*Flaviviridae* 31.5%, *Parvoviridae* 21.0%, *Siphoviridae* depletion to 17.4%; Fig. 2b). A comparison between the HIV viremic and non-viremic groups showed that the former was composed of 67.1% *Parvoviridae* and 6% *Flaviviridae*, while the latter comprised 2% par and 41.9% Fla. Furthermore, within the non-viremic group, *Flaviviridae* represented the predominant component in both the medium-CD4 and high-CD4 subgroups, accounting for 46.9% and 33.5%, respectively (Supplementary Fig. 2a, b).

Eukaryotic virus composition diverged radically between groups (Fig. 2c). Controls maintained balanced *Anelloviridae* (38.3%), *Flaviviridae* (31.8%), and *Picobirnaviridae* (18.4%) representation. HIV-detected individuals shifted to *Parvoviridae* dominance (77.0%), whereas HIV-undetected cases showed anomalous *Flaviviridae* inflation (72.9%) with depressed *Anelloviridae* (14.4%) and *Parvoviridae* (3.6%).

Throat viromes displayed contrasting stability (Fig. 2d–f). UPGMA trees revealed interspersed clustering between HIV-infected and control groups, reflecting conserved phage dominance (*Siphoviridae* 69.1%, *Myoviridae* 15.3%, *Podoviridae* 13.4% vs. 69.1%, 13.7%, 10.4% in controls). Eukaryotic components showed nuanced shifts: *Retroviridae* increased progressively from controls (17.7%) to HIV-detected (23.0%) and HIV-undetected (34.9%) groups, while *Parvoviridae* declined inversely from controls (25.0%) to HIV-detected (23.5%) and HIV-undetected (12.9%) cohorts. Additionally, the HIV-undetected cohort uniquely accumulated *Reoviridae* (11.4%) alongside diminished *Anelloviridae* (15.6%). Viral composition analyses conducted from the dual perspectives of HIV viral load and CD4 count both indicated that, whether comparing the viremic and non‑viremic groups or analyzing the medium‑CD4 versus high‑CD4 subgroups within the non‑viremic group, the viral profiles closely resembled that of the healthy control group, with *Siphoviridae* overwhelmingly dominating the composition (Supplementary Fig. 2c, d).

**Fig. 3:**
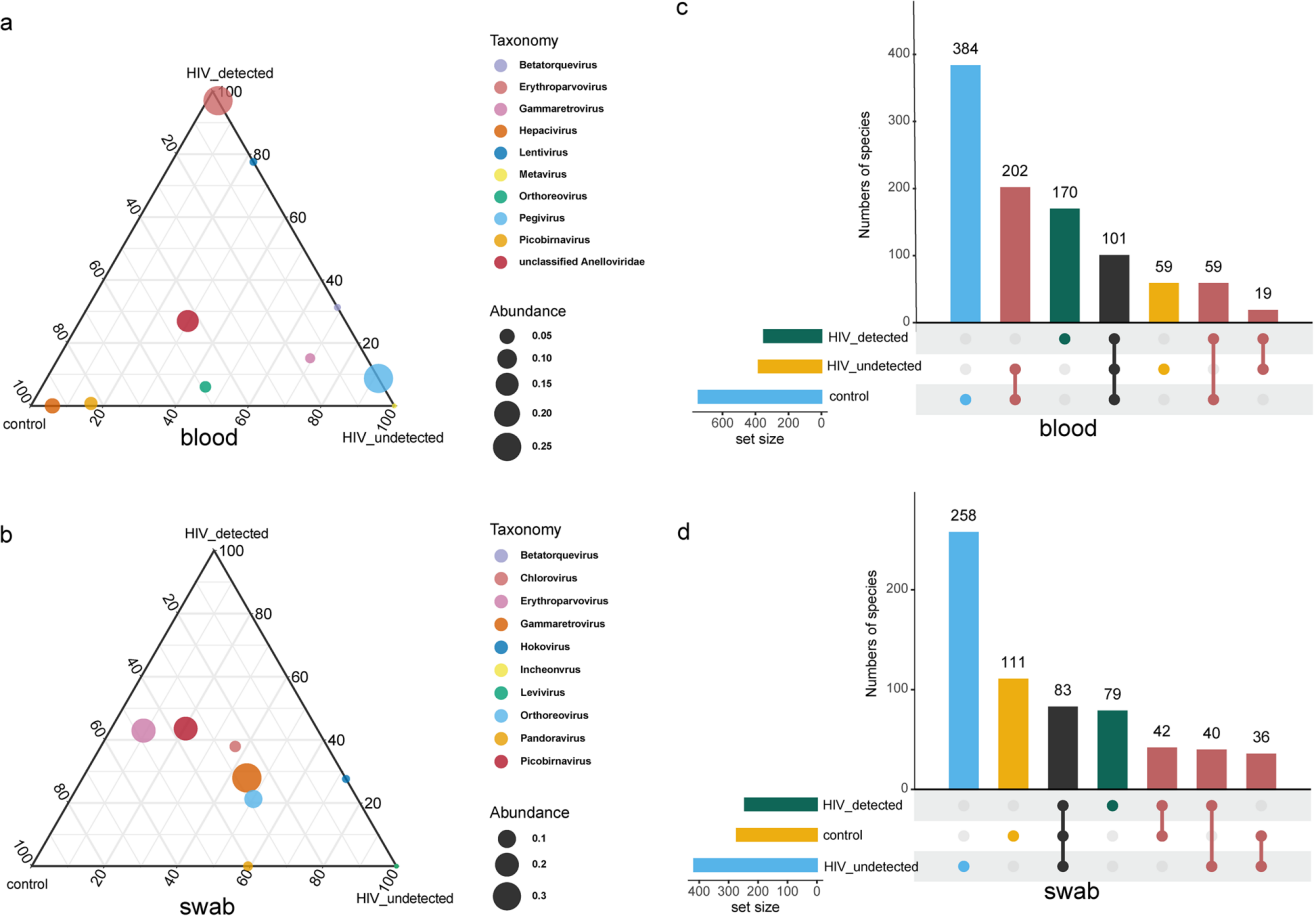
The composition of shared and unique viruses at the genus and species levels among the samples. **a** The ternary plot displays the relative abundance of the top ten eukaryotic virus genera in blood samples from HIV‑infected individuals, non‑infected individuals, and controls. Circle size is proportional to the abundance of each viral taxon, and different colors denote distinct viral families. **b** Depicts the relative abundance of the top ten eukaryotic virus genera in swab samples from the same three groups (HIV‑infected, non‑infected, and controls). **c**, **d** UpSet diagrams are used to show the numbers of overlapping and unique viral species among the three groups in blood and swab samples, respectively. Solid dots linked by vertical lines mark the intersections of shared species, whereas open light gray circles represent species that do not belong to those intersections. The vertical bars indicate the count of viral species within each intersection, and the horizontal bars display the total number of viral species in each group.

In genus-level profiling of eukaryotic viruses, blood viromes showed distinct HIV-associated stratification (Fig. 3a). The relative abundance of Erythroparvovirus demonstrated treatment-phase dependency: absent in controls, it surged to dominance in viremic patients (78.2%) before collapsing in aviremic individuals (2.4%). Meanwhile, Pegivirus displayed an inverse trajectory, escalating from negligible levels (7.0%) in HIV-detected cases to overwhelming prevalence (73.3%) during viral suppression. suggesting ART-mediated competitive displacement. Anelloviruses loads remained stable across groups. Throat virome succession patterns revealed three ART-linked transitions (Fig. 3b): Gammaretrovirus loads escalated from 29.2% (controls) to 48.7% (HIV-undetected), potentially facilitated by CD4^+^ T-cell depletion; Erythroparvovirus inversely declined from 32.8 to 6.3%, paralleling blood compartment reductions; and Orthoreovirus exhibited compensatory expansion (16.6% in HIV-undetected vs. 9.5% controls), coinciding with reduced *Anelloviridae* and *Parvoviridae* loads. These coordinated changes imply cross-compartmental ecological trade-offs during therapeutic intervention, where viral suppression in one niche may inadvertently fuel opportunistic colonization in others.

Deep sequencing resolved striking disparities in viral species richness across compartments and clinical groups (Fig. 3c, d). Blood specimens harbored 994 identifiable species, with controls exhibiting nearly double the richness of HIV-detected cohorts (746 vs. 349–381 species). This depletion may correlated with HIV progression stage, suggesting either therapeutic suppression or CD4^+^ lymphopenia constrains viral colonization. Core species (viral taxa that are consistently detected across the vast majority of samples) analysis identified 101 conserved blood viruses across all groups, likely representing persistent human virome constituents, while HIV-specific groups showed progressive niche specialization: 384 control-unique species versus 170/59 in HIV-detected/undetected groups. The throat virome identified 649 definitive viral species and demonstrated inverse ecological rules, with HIV-undetected individuals paradoxically retaining the highest unique species count (258 vs. 111 in controls). While core species conservation remained stable (*n* = 83), that the overlap between the control and HIV-undetected groups was 125 species, the sharing between the control and HIV-detected groups was 119 species, and the sharing between the HIV-detected and HIV-undetected groups was also 125 species.

### Diversities of the virome

**Fig. 4:**
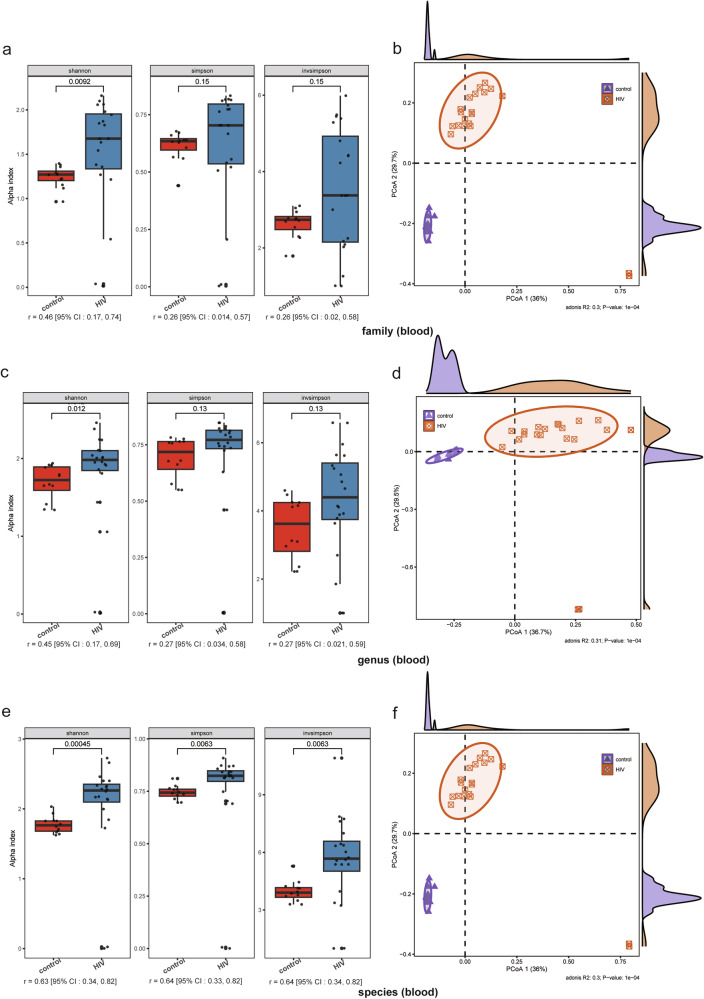
Analyses of viral diversities of the blood samples between healthy controls and the HIV infected groups. **a**, **c**, **e** The alpha diversities at the family, genus, and species levels were analyzed between the healthy control group (*n* = 12 pools) and the HIV‑infected group (*n* = 20 pools), based on the Shannon, Simpson, and inverse Simpson indices, respectively. **b**, **d**, **f** Beta diversity comparisons at the family, genus, and species levels were evaluated between the healthy control and HIV‑infected groups. Each ellipse and triangle denotes an individual library, and the extent of separation reflects differences in community composition. In addition, the distribution density of samples within each group is displayed, and the density curves on the top and left margins allow visualization of how the two groups are distributed in the PCoA space. The *R*² value ranges from 0 to 1, with values closer to 1 indicating a greater ability of the model to explain variation in community structure.

We employed multi-scale ecological metrics (Shannon index (H’) for species diversity, Simpson index (*λ*) for dominance concentration and inverse Simpson index (1/*λ*) for effective diversity) to quantify HIV-associated virome restructuring (Fig. 4a, c, e). At the family level, blood viromes from HIV-infected individuals exhibited significantly elevated Shannon diversity (H’, *p* = 0.0092), though Simpson indices showed only marginal increases; this pattern suggesting enhanced viral diversity in HIV-infected individuals. This diversification showed similarity at the genus-level analyses revealed universal metric increases (H’, *p* = 0.012). While the diversification intensified at finer resolutions, species-level evaluations confirmed concurrent richness and evenness enhancement (H’, *p* = 0.00045; *λ*, *p* = 0.0063; 1/*λ*, *p* = 0.0063).

UniFrac-based β-diversity analysis exposed fundamental architectural shifts, with HIV-infected blood viromes forming distinct PCoA clusters across all taxonomic levels. Notably, β-dispersion homogeneity tests revealed comparable effect sizes regardless of classification granularity (across family through species), implying HIV disrupts viral communities through broad ecological mechanisms rather than lineage-specific interactions. Although our observed diversity increase contrasts with typical microbiome depletion paradigms, this may reflect HIV’s unique capacity to enable opportunistic viral colonization while suppressing bacteriophage regulation (Fig. 4b, d, f). Moreover, neither the comparison between viremic and non‑viremic groups nor the diversity analysis of the medium‑CD4 and high‑CD4 subgroups within the non‑viremic group revealed significant differences in alpha diversity, and beta diversity likewise showed no clear separation between groups (Supplementary Fig. 3).

**Fig. 5:**
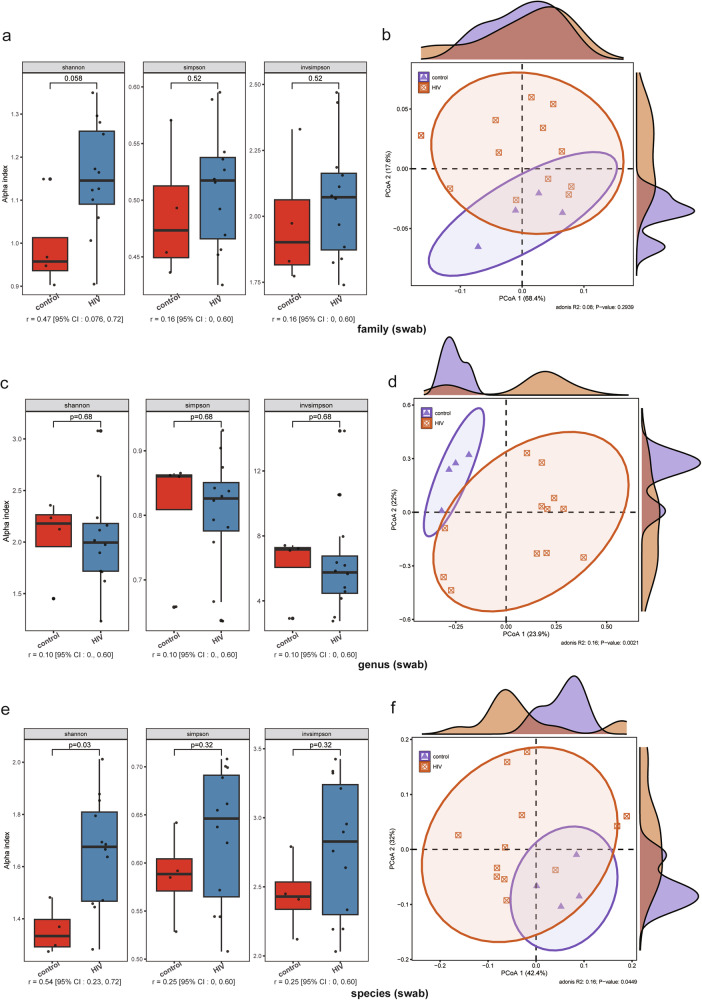
An analysis of viral diversity in throat swab samples was conducted between the healthy control group and the HIV-infected group. **a**, **c**, **e** Present the comparisons of alpha diversity at the family, genus, and species levels between the healthy control group (*n* = 4 pools) and the HIV‑infected group (*n* = 12 pools), as measured by the Shannon, Simpson, and inverse Simpson indices, respectively. **b**, **d**, **f** Assess beta diversity between the two groups across the same taxonomic levels. In these panels, envelopes and triangles denote individual sample libraries, and the spatial separation between groups reflects their compositional dissimilarity. The axes PCoA 1 and PCoA 2 represent the percentages of variation explained by each principal coordinate. Additionally, the density distributions of samples within each group are visualized, with marginal density curves on the top and left illustrating the spread of the two groups in PCoA space.

Throat virome analysis revealed distinct HIV-associated diversification patterns contingent on taxonomic resolution. While family-level metrics showed marginal, nonsignificant diversity increases in HIV-infected individuals, on the contrary, at the genus level, the diversity in the control group showed a marginal, nonsignificant increase. Species-level profiling uncovered significant Shannon index elevation (H’, *p* = 0.03) with borderline Simpson metric shifts—a dichotomy suggesting HIV preferentially amplifies rare/low-abundance species rather than restructuring dominant taxa. This taxonomic-scale dependency contrasts sharply with blood virome patterns, highlighting mucosal virome stability against HIV-driven ecological pressures (Fig. 5a, c, e). β-Diversity trajectories exhibited parallel resolution-dependent effects. Family-level PCoA failed to clearly distinguish between HIV-infected and control groups, while genus-level analysis exposed distinct clustering (*R*² = 0.16, *p* = 0.021) that partially dissipated at species resolution. Notably, HIV-infected cohorts consistently demonstrated expanded β-dispersion across all taxonomic tiers, indicating sustained virome architectural instability despite antiretroviral control (Fig. 5b, d, f). Mirroring the findings from the HIV versus control comparison, the medium‑CD4 and high‑CD4 groups exhibited only subtle, nonsignificant differences in diversity at the family level (Supplementary Fig. 4).

### Difference of virome

**Fig. 6:**
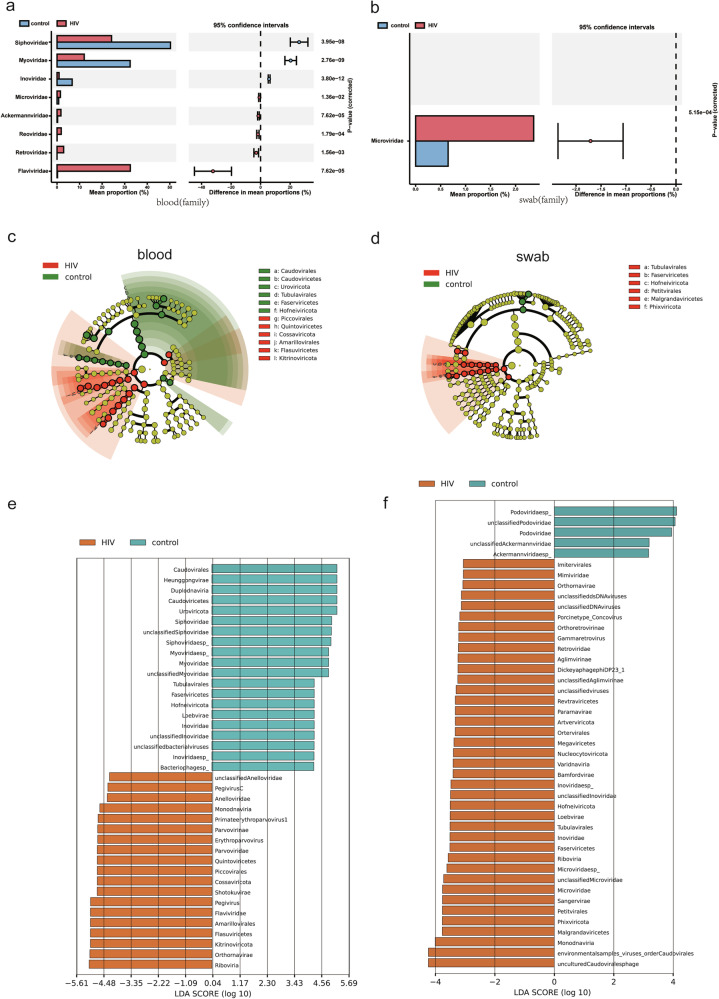
Differential analysis and identification of potential biomarkers. **a**, **b** Differential analysis of the virome at the family level was performed using STAMP for blood samples (control: 12 pools; HIV‑infected: 20 pools) and throat swab samples (control: 4 pools; HIV‑infected: 12 pools), respectively. In each panel, bar charts illustrate the relative abundance of individual species across the different groups. **c**, **d** LEfSe analysis was carried out on blood and throat swab samples, respectively. In the taxonomic cladogram, each concentric circle corresponds to a distinct phylogenetic rank. Colored circles denote groups in which the indicated taxa are more abundant relative to other groups, whereas yellow circles indicate non‑significant differences. Taxa showing significant differences are listed to the right of the cladogram. **e**, **f** Linear discriminant analysis (LDA) was applied to blood and throat swab samples, respectively. A bar graph displays the taxa with the most pronounced abundance differences, based on their LDA scores (log10) as an estimate of effect size. Only taxa meeting the criteria of a *P* < 0.05 and an LDA score above the significance cut‑off (4 for blood, 3 for throat swab) are presented.

To identify differentially abundant viral taxa across taxonomic ranks, we performed comparative metagenomics using STAMP v2.1.3 with rigorous statistical validation. Two-group comparisons (HIV-detected patients vs controls) employed Welch’s unequal variance t-test followed by Benjamini–Hochberg FDR correction (significance threshold: *P*adj < 0.05). Blood virome analysis at the family level revealed eight differentially abundant taxa. HIV-detected patients showed significant Flaviviridae enrichment (*P*adj = 7.62e-05), contrasting with control-specific *Siphoviridae* (*P*adj = 3.95e-08) and *Myoviridae* (*P*adj = 2.76e-09) predominance (Fig. 6a).

The medium‑CD4 and high‑CD4 groups diverged markedly in viral family abundance, with the former showing significant *Parvoviridae* (*P*adj = 3.43e-02) enrichment and the latter dominated by *Retroviridae* (*P*adj = 1.52e-02) (Supplementary Fig. 5).

Throat microbiomes exhibited narrower inter-group variation, with *Microviridae* being the sole significantly enriched family in HIV-infected subjects (*P*adj = 5.15e-04; Fig. 6b).

For biomarker discovery, we integrated LEfSe (LDA threshold >4.0 for blood sample, LDA threshold >3.0 for throat swab sample) with nonparametric testing (Kruskal–Wallis/Wilcoxon, *p* < 0.05). Blood viromes demonstrated stark phage compositional shifts: in the control group’s blood virome, Caudoviricetes and its *Myoviridae*, *Siphoviridae*, and *Podoviridae* families showed significant LDA scores. HIV-infected blood specimens exhibited distinct viral signatures: Cossaviricota phylum (containing Quintoviricetes class, Piccovirales order, *Parvoviridae* family, and *Erythroparvovirus* genus, primate erythroparvovirus1 (B19V) species) and Kitrinoviricota phylum (including Flasuviricetes class, Amarillovirales order, *Flaviviridae* family, Pegivirus genus and pegivirus C species) showed significantly elevated LDA scores. Notably, the *Anelloviridae* family and unclassified *Anelloviridae* also displayed marked differential abundance. These patterns nominate parvovirus B19, pegivirus C, and Anelloviruses as candidate HIV progression biomarkers (Fig. 6c, e). Throat virome analysis revealed ecological niche-specific patterns (Fig. 6d, f). In controls, *Podoviridae* demonstrated the highest LDA score, while the HIV-infected group exhibited differential abundance of multiple phage taxa, with *Microviridae* showing predominant LDA scores, suggests *Microviridae* as potential pharyngeal HIV biomarkers.

### Species correlation

**Fig. 7:**
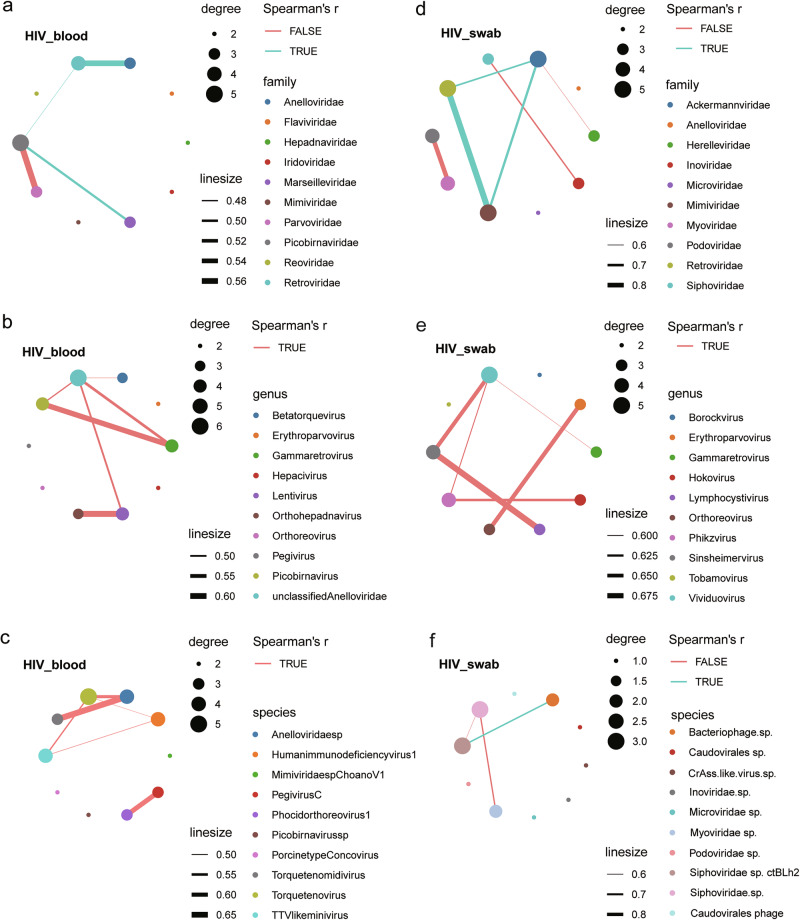
The correlations among viruses. **a**–**c** Respectively analyze the correlations among the top ten most abundant viruses at the family, genus, and species levels in blood samples from HIV-infected individuals. **d**–**f** Respectively analyze the correlations among the top ten most abundant viruses at the family, genus, and species levels in throat swab samples from HIV-infected individuals. The size of the nodes represents the number of connections each virus at the respective taxonomic level has with other viruses. The greater the degree, the larger the node. Spearman’s rank correlation coefficient (edge color): the color of the edges represents the Spearman rank correlation coefficient, which is used to measure the correlation between two variables. Red (FALSE) indicates a negative correlation or no significant correlation. Blue (TRUE) indicates a positive correlation. Line thickness (thickness of edges): the thickness of the edges indicates the magnitude of the Spearman correlation coefficient. Different colors represent different viruses.

Characterization of eukaryotic viral correlations in blood samples from HIV-infected individuals revealed compartment-specific interaction networks across taxonomic hierarchies. At the family level, *Picobirnaviridae* emerged as the most interconnected viral family, displaying the highest network connectivity. This family exhibited distinct association patterns, including a significant inverse relationship with *Parvoviridae* (Spearman’s *r* = −0.568) and positive correlations with *Marseilleviridae* (*r* = +0.499) and *Retroviridae* (*r* = +0.462). Notably, the robust positive covariation between *Retroviridae* and *Anelloviridae* (*r* = +0.562) suggests potential immune-mediated co-persistence mechanisms (Fig. 7a).

Genus-level network analysis identified unclassified *Anelloviridae* as a central topological hub, demonstrating coordinated abundance fluctuations with multiple genera. Significant positive associations were observed with gammaretrovirus (*r* = +0.524), lentivirus (*r* = +0.503), and picobirnavirus (*r* = +0.488). The strong gammaretrovirus–picobirnavirus interaction (*r* = +0.591) and orthohepadnavirus-lentivirus linkage (*r* = +0.608) may reflect shared cellular tropism for CD4^+^ T-lymphocyte subsets (Fig. 7b). Species-level analysis revealed three key associations: torquetenovirus displayed co-occurrence patterns with both TTV-like minivirus (*r* = +0.570) and HIV-1 (*r* = +0.483), while pegivirus C demonstrated marked covariation with Phocid orthoreovirus 1 (*r* = +0.622) (Fig. 7c).

In oropharyngeal samples, hierarchical interaction patterns differed substantially from blood compartment observations. Family-level analysis uncovered a pronounced *Ackermannviridae-Herelleviridae* anticorrelation (*r* = −0.587), contrasting with the extreme negative *Retroviridae-Mimiviridae* association (*r* = −0.874). At the genus level, vividuovirus exhibited synchronized abundance patterns with sinsheimervirus (*r* = +0.655), lymphocystivirus (*r* = +0.691), and orthoreovirus (*r* = +0.661). Species-specific dynamics included antagonistic relationships between *Siphoviridae* sp. and *Myoviridae* sp. (*r* = −0.657), alongside positive *Siphoviridae* sp. ctBLh2-Bacteriophage sp. covariation (*r* = +0.671).

These findings highlight anatomical compartment-specific viral interaction networks in HIV infection, potentially driven by immune selection pressures and ecological niche competition. The consistent centrality of *Anelloviridae* across multiple anatomical sites raises important questions regarding its potential role as a modulator of viral community dynamics, warranting further mechanistic studies on cross-viral regulatory mechanisms in immunocompromised hosts.

### Identification and phylogenetic analysis of viruses

To investigate the evolutionary characteristics of key viruses within the virome of HIV patients under ART pressure, including dominant constituents and pathogenic viruses, we obtained the corresponding viral sequences and conducted phylogenetic analysis to construct evolutionary trees. The key virological findings are detailed below by category.

**Fig. 8:**
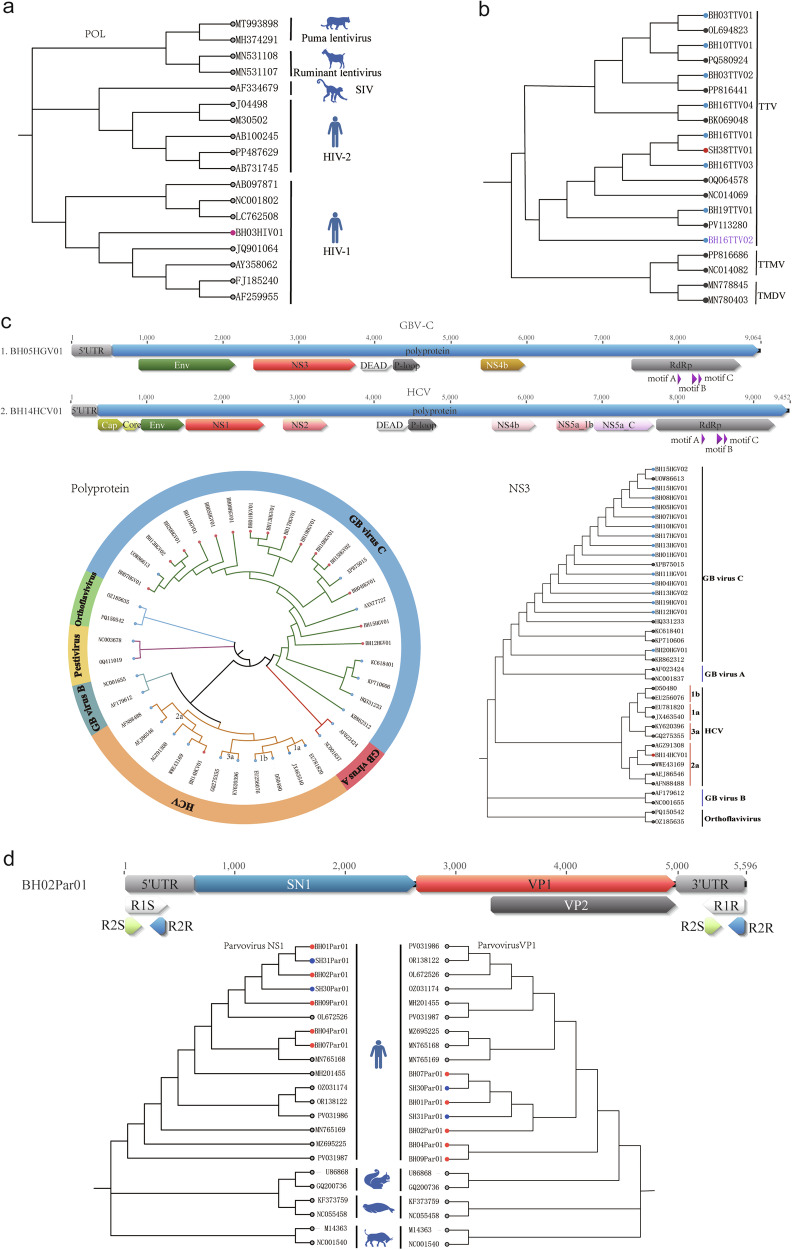
Viral structure and evolution analysis. **a** Evolutionary analysis was conducted using the pol gene of HIV, with red circles indicating the viral sequences obtained in this study. **b** The phylogenetic tree was constructed based on the ORF1 nucleotide sequences of anelloviridae, with blue annotations indicating the viral sequences obtained from blood samples, and red circles indicating those from throat swab samples. **c** The upper panel shows the genomic structure and functional annotation of GBV-C and HCV obtained in this study. The left circular phylogenetic tree below is constructed based on the polyprotein, with red markings indicating the sequences obtained in this study; the right phylogenetic tree is constructed based on the NS1 protein sequences, with blue markings indicating the sequences obtained in this study. **d** The upper panel displays the full genomic structure and functional annotation of the parvoviruses obtained in this study, with R1S (1–383nt) and R1R (5214–5596nt) being complementary sequences. The two R2S are repetitive sequences, as are the two R2R, and additionally, R2S and R2R are complementary sequences. The phylogenetic trees on the left and right below are constructed based on the NS1 and VP1 genes, respectively. Sequences obtained in this study are marked with red (blood) and blue (throat swab) circles. The viral genomic structures were generated using Geneious Primer V2025.0.2 software; the human and animal icons in the phylogenetic trees are used under license from Figdraw (license number: ID:ROPUAc4998).

We acquired a 4170 bp HIV-1 contig from one sequencing pool. This contig spans the complete pol gene (designated BH03HIV01). Phylogenetic analysis placed this isolate within the HIV-1 CRF07_BC recombinant cluster, sharing 95.6% nucleotide identity with the subtype B reference strain JQ901064 (Fig. 8a).

*Anelloviridae* family encompasses four major human-tropic genera: alphatorquevirus, betatorquevirus, gammatorquevirus, and hetcovirus^24^. We obtained nine sequences (2774–3728 bp), each containing a complete ORF1, predominantly from HIV-infected blood samples (8/9), and one from a HIV-infected individual's throat swab sample. Phylogenetic reconstruction clustered these sequences within alphatorquevirus, showing >90% identity to NCBI reference sequences (Fig. 8b).

Human pegivirus C (GBV-C, 9.4 kb) and Hepatitis C virus (HCV, 9.6 kb) are positive-sense RNA viruses within *Flaviviridae*^25,26^. Parallel sequencing of HIV-positive sera identified 15 pegivirus genomes (8.4–9.1 kb) and one HCV genotype 2a genome (9452 bp). This HCV isolate encodes a complete polyprotein (3010 aa) and clusters closely (96.4% identity) with a previously reported strain (PP372686) (Fig. 8c). Pegivirus sequences grouped monophyletically with GBV-C strains, and NS3 protease phylogeny mirrored polyprotein topology, indicating conserved evolution.

Human parvovirus B19 (B19V) is a common respiratory-transmitted pathogen, often infecting children and potentially causing chronic anemia in immunocompromised hosts^27^, and may cause chronic anemia or persistent viremia in immunocompromised individuals^28^. In this study, seven complete B19V genomes were obtained from HIV-positive individuals. Phylogenetic analysis of concatenated NS1-VP1 sequences placed all isolates within the canonical human parvovirus clade (>96% identity to closest homologs) (Fig. 8d).

Picobirnavirus (PBV) is epidemiologically associated with acute watery diarrhea and gastroenteritis, and current evidence suggests it may act as an opportunistic pathogen in immunocompetent hosts with potentially increased virulence in immunocompromised individuals^29^. In this study, human picobirnavirus sequences were identified in respiratory samples, comprising two capsid protein genes (1734 bp and 2224 bp, the latter a complete CDS) and three RdRp genes (1489 bp, 1512 bp, and 1601 bp). These sequences showed 78.4–97.3% nucleotide similarity to their closest database homologs. Meanwhile, a full-length HBV subtype C genome was successfully assembled from a blood sample. Additionally, Brisavirus sequences were detected in respiratory samples, comprising a 1221 bp partial Cap gene and a 1112 bp complete Rep gene; this Brisavirus is epidemiologically associated with severe clinical conditions and periodontitis^30^. Phylogenetic analysis was performed on all viral sequences obtained above, similarities exceeding 97% to their closest homologs in the public database (Supplementary Fig. 6).

### Functional enrichment analysis of the virome genome

Functional differential gene analysis and gene set enrichment analysis (GSEA) were performed based on InterPro domain annotations (INTERPRO2GO) to investigate functional alterations in the virome of HIV patients. The results revealed differentially expressed genes between the HIV patient group and healthy controls in blood samples. Among the 292 upregulated genes, significantly elevated expressions were observed for Major surface glycoprotein G, Influenza virus matrix protein 2, and RNA-dependent RNA polymerase (hepatitis). In contrast, among the 2271 downregulated genes, notable reductions were detected in solute-binding protein family 5, TRAP transporter large membrane protein, and quinolinate synthetase A (Supplementary Fig. 7a).

GSEA identified eight significantly enriched upregulated GO terms (Supplementary Fig. 7b) and one downregulated GO term (FDR < 0.05) (Supplementary Fig. 7c). No significantly enriched GO terms were observed between the HIV_undetected and HIV_detected groups. In pharyngeal swab samples, no GO terms reached the significance threshold in either the comparison between HIV patients and healthy controls, or between HIV_undetected and HIV_detected groups.

## Discussion

Our comparative analysis of blood and oropharyngeal viromes in ART-treated HIV patients versus healthy controls reveals critical HIV-associated virome alterations with clinical implications. The findings highlight systemic viral community restructuring in HIV patients through taxonomic shifts, modified diversity patterns, and unique viral interactions, while identifying potential biomarkers for clinical monitoring.

The blood virome transition from bacteriophage predominance (89.3% Caudovirales in controls) to eukaryotic viral dominance (52.5% *Flaviviridae/Parvoviridae* in HIV^+^ group) reflects HIV-induced immune dysregulation. Three mechanisms likely drive this shift: First, compromised CD8^+^ T-cell surveillance enables opportunistic viral proliferation^31^. Second, ART-mediated partial immune reconstitution creates permissive conditions for chronic coinfections despite CD4^+^ T-cell recovery^32^. Third, disrupted bacteriophage predation reduces ecological competition for eukaryotic viruses^33^. Notably, *Parvoviridae* dominance in samples BHIV02/BHIV04 aligns with parvovirus B19,s erythroid progenitor cell tropism, granting it a replicative advantage in immunocompromised hosts^34^.

GBV-C, a blood-borne virus within the family *Flaviviridae*, shows high prevalence among individuals infected with HIV-1 and/or HCV^35^. GBV-C enrichment in HIV patient blood specimens correlates with GBV-C’s immunomodulatory properties^36^. GBV-C has a broad tropism for lymphoid and myeloid cells (including CD4^+^ T cells) and targets the same host cell types as HIV-1^37^. Mechanistic studies propose that elevated GBV-C loads may suppress HIV replication through immune pathway modulation^38^. This viromic dominance likely stems from GBV-C’s adaptation to HIV-associated immunosuppression, where it alters host responses and viral competition dynamics^39^. In this study, blood virome analysis of HIV-infected individuals receiving ART revealed significant expansion of *Flaviviridae* (primarily GBV-C) and *Anelloviridae*, Moreover, both the species richness and Shannon diversity index of the viral community were higher than those in the healthy control group, suggesting that ART is still insufficient to fully reverse the homeostatic imbalance in the plasma virome caused by HIV infection, a finding consistent with previously reported longitudinal results. However, our study observed a distinct compositional distribution of eukaryotic viruses in the HIV-undetectable group: *Flaviviridae* accounted for 72.9%, while *Anelloviridae* constituted 14.4%. This differs from the reported pattern in which *Anelloviridae* was the predominant eukaryotic virus family in blood (97.1%), followed by *Flaviviridae* (2.04%)^13^. Although most studies indicate that the blood virome of HIV-infected individuals receiving ART is predominantly composed of *Anelloviridae*, *Flaviviridae* can also emerge as the dominant viral family in some individuals^40,41^. Furthermore, ART treatment significantly reduces the load of *Anelloviridae* while increasing the relative proportion of *Flaviviridae*^13^. While effectively suppressing HIV, ART may promote GBV-C replication by reducing inter-viral competition; the observed increase in its abundance is more likely attributable to the reactivation of latent virus within the host. Moreover, the over-expansion of GBV-C may further suppress the replication of *Anelloviridae*.

GBV-C appears to exert a “double-edged sword” effect. Conventional understanding holds that GBV-C coinfection may confer a protective role against HIV disease progression by inhibiting HIV assembly, downregulating HIV co-receptors (such as CCR5 and CXCR4), upregulating levels of chemokines including RANTES, MIP-1α, MIP-1β, and SDF-1, and attenuating immune activation^42–44^. However, emerging evidence suggests that in the ART era, the expansion of GBV-C may reflect inadequate immune containment rather than a protective effect^13^.

Human cytomegalovirus (CMV) and Epstein–Barr virus (EBV), both double-stranded DNA viruses belong to the Herpesviridae family, are clinically significant opportunistic pathogens traditionally prevalent among individuals with HIV/AIDS and other immunocompromised states^45^. Studies indicate that these viruses not only act as co-factors accelerating HIV disease progression but also serve as drivers exacerbating the clinical manifestations of other opportunistic infections^46,47^.

ART effectively suppresses HIV replication and facilitates the restoration of immune system function, particularly through the recovery of CD4⁺ T-cell numbers and activity, a process termed “immune reconstitution.” The reconstituted immune system significantly enhances immune surveillance and control over latent herpesviruses, such as EBV and CMV, thereby reducing their reactivation frequency and lowering the likelihood of detecting viral DNA in peripheral blood^47,48^. In the present study, the detection rates of CMV and EBV in the plasma of HIV patients were consistent with previous reports: only 4 cases were CMV positive (4/203) and 3 cases were EBV positive (3/203). The metagenomic analysis also did not detect expansion of CMV or EBV. Although the detection rate of cell-free EBV DNA in the peripheral blood of individuals receiving ART is generally low, a separate virome study revealed a distinct pattern: compared to the control group, the relative abundance of EBV was significantly higher in the plasma of HIV-infected individuals on long-term ART^49^. EBV can infect various immune cells, including monocytes, T cells, and B cells^50^, and activates the NF-κB signaling pathway through its LMP-1 gene, thereby promoting TNF-α secretion^51,52^. In immunocompromised individuals or those with HIV infection, a high EBV load may increase the risk of lymphoma through TNF-mediated polyclonal B-cell activation, triggered directly by HIV-1 proteins or indirectly via TLR recognition of microbial products^53,54^. Studies have shown that despite effective ART-mediated immune preservation, EBV load is not reduced and continues to be associated with LPS and pro-inflammatory cytokines (IL-6, IL-10, and TNF-α) in these patients^49,55^.

Contrary to expectations of reduced diversity in immunodeficiency, HIV-infected blood viromes showed increased α-diversity (Shannon index)^56^. This paradox may arise from: (1) ART-induced partial immune recovery, facilitating viral coinfections^57,58^. (2) Heightened replication of commensal viruses like Anelloviruses in immunocompromised hosts^40^. (3) Reactivation of latent viral reservoirs amplifying community complexity^59,60^. Similarly, analogous virome alterations are observed in the gut, mirroring those seen in the blood: severe depletion of CD4⁺ T cells in gut-associated lymphoid tissue impairs mucosal immune surveillance, driving a marked expansion of opportunistic eukaryotic viruses, such as *Anelloviridae* and *Adenoviridae*, alongside a reduction in bacterial populations and specific bacteriophage taxa. Moreover, intestinal abundance of *Anelloviridae* exhibits an inverse correlation with CD4⁺T-cell counts and independently predicts poor immune recovery following antiretroviral therapy^14,61^. This cross-site consistency indicates that the expansion of anelloviruses reflects a generalized response to systemic immune compromise rather than being confined to a specific organ. Thus, it may serve as a universal biomarker indicative of a state of immune “debt” in the host.

Clinically relevant biomarkers identified through LEfSe analysis include *Erythroparvovirus*, pegivirus C, and *Anelloviruses*. Among eukaryotic viruses, the elevated abundance of *Erythroparvovirus* (B19V) (78.2% in the viremic group) corresponds to its established tropism for erythroid precursor cells^34^. The strong association of pegivirus C with the HIV-detectable cohort (73.3%) reinforces its immunomodulatory properties, which may attenuate HIV disease progression via interferon-gamma (IFN-γ) induction^62^. The positive correlation between *Anelloviruses* and HIV (*r* = +0.503) reinforces TTV’s potential as an immune status biomarker, which aligns with prior evidence^63^. Viral interaction networks reveal ecological drivers: Picobirnaviridae’s central network position and Anelloviridae’s hub status suggest niche-modifying capabilities. *Retroviridae-Anelloviridae* covariation (*r* = +0.562) may reflect shared latency strategies^64^.

HIV infection can alter the lung microbiome and may be associated with chronic lung diseases^65^, yet the role of the upper respiratory tract virome as an “upstream sentinel” remains unclear. This study found that in ART-treated people with HIV, the oropharyngeal virome exhibited the following characteristics: subtle shifts in the eukaryotic viral component (e.g., *Retroviridae*, *Parvoviridae*), while bacteriophages (particularly *Siphoviridae*) remained highly stable. This mirrors reports that the lung bacterial microbiome can partially normalize following effective ART^66^. The structural instability of the oropharyngeal virome (manifested as increased β-dispersion) may reflect and contribute to local chronic immune activation, compromising the mucosal barrier and promoting the micro-aspiration or translocation of upper respiratory pathogens to the lungs, analogous to the gut microbial translocation observed in HIV infection^67^. Critically, the abundance of *Retroviridae* (which includes endogenous retroviruses) showed a progressive increase in the oropharynx of people with HIV. Retroviral elements are known to stimulate innate immunity, drive interferon signaling pathways, and exacerbate local and even lower respiratory tract inflammation^68^. There exists a direct ecological link between the oropharyngeal virome and lung health. Studies on the lung microbiome have confirmed that colonization by oral-origin bacteria is associated with enhanced inflammation^20,69^. By analogy, an increase in specific viral taxa or alterations in virus-virus interactions within the oropharyngeal virome may affect the stability of the entire respiratory microbial network through viral-bacterial interplay. The dysregulated fluctuations in the diversity of the upper respiratory tract virome post-ART suggest that it is not merely a reflection of pulmonary dysbiosis but may also serve as an independent factor driving chronic lung inflammation and infection risk in people with HIV. Although the study analyzed the oropharyngeal virome by controlling for confounding factors, such as age, sex, season, and CMV infection, the results remain constrained by the fact that the virome itself is collectively influenced by multiple factors, making it difficult to fully reveal the true differences and associations.

This study has several limitations. First, the cross-sectional design limits the ability to track long-term immune evolution or individual treatment histories, thus requiring longitudinal studies for further clarification. Second, despite strict contamination controls, background interference cannot be completely excluded, including possible residual contamination from sources, such as reagents or the environment. Third, while sample pooling improved sequencing depth and cost-effectiveness, it may mask inter-individual heterogeneity, hindering the identification of sample-specific signals. Finally, due to unavailable clinical and behavioral data, the potential impacts of confounders (e.g., medications, substance use, diet) on the virome could not be assessed^70,71^.

In summary, HIV infection triggers a multi-site “viral ecological revolution” within the human body, leading to profound remodeling of the viromes in the gut, blood, lungs, and respiratory tract. This constitutes a shared landscape of dysbiosis, painted collectively by immunodeficiency. The alterations in viromes across these sites are not isolated events; instead, they interact by driving local and systemic inflammation, collectively forming the microbiological basis for the chronicity of HIV disease and the development of its comorbidities. Future research needs to delve deeper into the mechanisms of direct interactions among these viromes (e.g., through immune cells or circulating factors) and adopt integrated analyses of multi-site viromes, thereby providing a more comprehensive perspective for developing novel microbiome-based diagnostic tools and intervention strategies.

## Methods

### Participant information

This study included a total of 203 HIV-1-infected individuals (164 males, 39 females). All enrolled individuals with HIV were confirmed to be on continuous ART for ≥1 year. Participants assigned to the HIV undetected group were required to have undetectable HIV RNA for ≥6 consecutive months. Additionally, 120 HIV-1-negative healthy individuals (94 males, 26 females) were included as the control group. All participants were aged between 18 and 55 years (Table 1).

### Ethics approval

This study adhered to the ethical principles of the Declaration of Helsinki. Written informed consent was obtained from all adult participants. Protocols for sample collection and experimental procedures were approved by the Ethics Committee of Taizhou People’s Hospital (KY2024-02b-01).

### Sample collection and preparation

Blood samples (203 from patients and 120 from controls) and throat swab samples (169 from patients and 64 from controls) were collected from 203 antiretroviral-treated HIV-infected individuals and 120 healthy controls in 2019. From each plasma sample, 200–500 µL was accurately aliquoted into 1.5 mL RNase-free microcentrifuge tubes. Throat swab samples were collected and transferred into 2 mL sterile EP tubes. All samples were stored at −80 °C until further analysis.

### Immune profiling and viral load quantification

CD4^+^ and CD8^+^ T-cell counts were determined using EDTA-anticoagulated whole blood analyzed on a BD FACSCalibur flow cytometer (gating strategy is shown in Supplementary Fig. 8), with CD4^+^/CD8^+^ ratios interpreted against the reference range (0.71–2.78). HIV viral loads were quantified via the Abbott m2000rt system (real-time quantitative PCR) and with its matching reagents: >40 copies/mL (quantifiable), <40 copies/mL (detectable but subquantifiable), or “undetectable” if no RNA was identified. Both CMV and EBV DNA levels were measured on the ABI 7500 platform using DAAV GEVE reagents, with a negativity threshold defined as <500 copies/mL.

### Sample preparation and viral library construction

Frozen specimens were thawed at 4 °C. Throat swabs were resuspended in 0.5 mL DPBS, vortexed twice (1800 rpm for 5 min each; 15-min intervals), and centrifuged (15,000 × *g*, 10 min, 4 °C) to pellet debri^72^. Clarified supernatants were pooled (*n* = 48 pools; 8–12 plasma or 10–16 swabs per pool; 30–50 µl supernatant per sample, stratified by health status, HIV viral load, and CD4^**+**^ T-cell counts). Pooled samples were filtered (0.45 μm pore membrane; Millipore) to eliminate residual cellular debris and particulate matter^73^. To selectively concentrate viral particles, we performed enzymatic digestion using a nuclease cocktail (TURBO DNase from Ambion with Cat. No. AM2239, Benzonase Nuclease from Merck with Cat. No. 70664-3, Baseline-ZERO from Epicentre with Cat. No. DB0715K and RNase A from Thermo Fisher Scientific with Cat. No. EN0531), 34 μl/pool at 37 °C for 60 min, effectively eliminating unprotected host and environmental nucleic acids^74^. Viral nucleic acids were extracted using the QIAamp MinElute Virus Spin Kit. cDNA synthesis used SuperScript III (Invitrogen, 18080093), 1 μl/pool and Klenow polymerase (NEB, M0210L), 1 μl/pool, followed by library preparation with Nextera XT Pre kit (Illumina, FC-131-1024) and paired-end sequencing (MiSeq, 2 × 250 bp). The sequencing quality statistics for each sample are summarized in Supplementary Data 3.

### Viral metagenomic raw data processing

Paired-end sequencing (250 bp read length) was performed on an Illumina MiSeq platform. Raw reads were demultiplexed using Illumina’s vendor-specific software (v2.6) for library-specific read assignment. Data preprocessing was executed via an upgraded in-house bioinformatics pipeline implemented on a high-performance Linux server (36 computational nodes). The pipeline included: adapter and quality trimming: automated removal of adapter sequences and low-quality bases (Phred score <30) using fastp (v0.23.4, default parameters)^75^. Host/bacterial sequence depletion: Bowtie2 (v2.4.5)^76^. alignment against composite reference genomes (human GRCh38 and bacterial RefSeq) with stringent filtering (mapping quality >30).

Viral sequences were identified through DIAMOND v2.1.8 using BLASTx alignment against the NCBI nonredundant (nr) protein database (*E*-value threshold <10^−^⁵). Following identification, paired-end reads were consolidated and taxonomically classified via MEGAN-CE v6.22.2. The data used in this analysis are presented in Supplementary Data 4–9. For de novo genome reconstruction, we implemented MEGAHIT v1.2.9.

### Statistics and reproducibility

To address sequencing depth variability, we generated normalized virome profiles from the 48 libraries using MEGAN’s rarefaction module. Subsequent multi-tiered statistical analysis was performed in R v4.4.1. Community α‑diversity was calculated using packages (e.g., vegan v2.6-4). For diversity comparisons, the Wilcoxon rank‑sum test (Mann–Whitney *U* test) was used, with effect size *r* computed as the rank‑biserial correlation, and confidence intervals estimated via nonparametric bootstrap (default resamples *R* = 500). Principal coordinate analysis (PCoA) based on unweighted UniFrac distances was applied to compare β-diversity, and the corresponding visualizations were generated using the ggpubr package (v0.6.0). STAMP v2.1.3 implemented Welch’s t-test with Benjamini–Hochberg FDR correction to identify HIV-associated viral taxa when compared to healthy controls. Viral co-occurrence patterns were resolved using the psych package’s correlation algorithms, while species overlap between cohorts was visualized through UpSetR v1.4.0. For age comparisons between groups, an independent samples t-test was employed, assuming normal distribution of data. Gender distribution was analyzed using Pearson’s chi-square test (*χ*²) to assess proportional differences. Predetermined significance threshold of *p* < 0.05. Biomarker potential was further evaluated through LEfSe analysis on the Galaxy platform.

### Phylogenetic characterization of viral lineages

For each viral lineage, we selected signature genomic regions to establish evolutionary relationships. HIV-1: protease-reverse transcriptase domains (pol gene); anelloviruses: capsid protein-encoding ORF1 (nucleotide-level); pegiviruses and HCV: full polyprotein sequences; parvoviruses: replication (NS1) and capsid (VP1) protein regions; picobirnaviruses: segmented structural (Cap) and polymerase (RdRp) phylogenies; HBV: complete genomic sequences; brisaviruses: bipartite Rep-Cap gene analysis. Multiple alignments were executed in MEGA11 using MUSCLE (gap penalty: -400), with maximum likelihood trees constructed in IQ-TREE (1000 ultrafast bootstraps; ModelFinder-optimized substitution matrices). The final phylogenetic trees were positioned based on the known strains matched in the NCBI database and were beautified through the iTOL platform.

### Quality control

To ensure sample integrity and prevent cross-contamination, all procedures, including sample preparation, nucleic acid extraction, library construction, and PCR amplification, were carried out in physically separated, dedicated laboratory spaces, in strict accordance with our established standard operating protocols. To safeguard against nucleic acid degradation, all consumables and surfaces were confirmed to be DNase- and RNase-free. Extracted nucleic acids were resuspended in DEPC-treated water (Sangon Biotech) supplemented with RNase inhibitors. In parallel, a blank control consisting of sterile ddH2O (Sangon Biotech) was processed under identical conditions to monitor for potential environmental, reagent, or procedural contamination. Furthermore, during the downstream analysis of the raw data, stringent abundance thresholds were applied to filter out low-abundance sequences potentially introduced by contamination, thereby mitigating background noise.

### Reporting summary

Further information on research design is available in the Nature Portfolio Reporting Summary linked to this article.

## Supplementary information

Supplementary Information

Description of Additional Supplementary Materials

Supplementary Data 1

Supplementary Data 2

Supplementary Data 3

Supplementary Data 4

Supplementary Data 5

Supplementary Data 6

Supplementary Data 7

Supplementary Data 8

Supplementary Data 9

Reporting Summary

## Data Availability

Raw sequencing data are deposited in the NCBI Sequence Read Archive under BioProjects PRJNA1256109^77^. Viral sequences are available in GenBank (accession nos. PV555108-PV555148). All Supplementary materials have been deposited in Figshare and are publicly available. The Supplementary data can be accessed via the following link (10.6084/m9.figshare.31048189)^78^.

## Code availability

All source code is publicly available on Zenodo (10.5281/zenodo.19777507)^79^. Further information is available from the corresponding authors upon reasonable request.
