# Supplementary material for: HIV-driven virome dysbiosis unveils distinct virome features and inter-viral correlations in blood and respiratory niches
Source: Commun Biol. 2026 May 8;9:958. doi: 10.1038/s42003-026-10221-z (PMC13369948; doi:10.1038/s42003-026-10221-z)
Supplement: Supplementary file 1 — Supplementary Information [file 42003_2026_10221_MOESM1_ESM.pdf]

Supplementary Figures with Legends

Supplementary Figure 1. Comparison of Immune Cell Counts Between HIV-detected patients and HIV-undetected individuals.

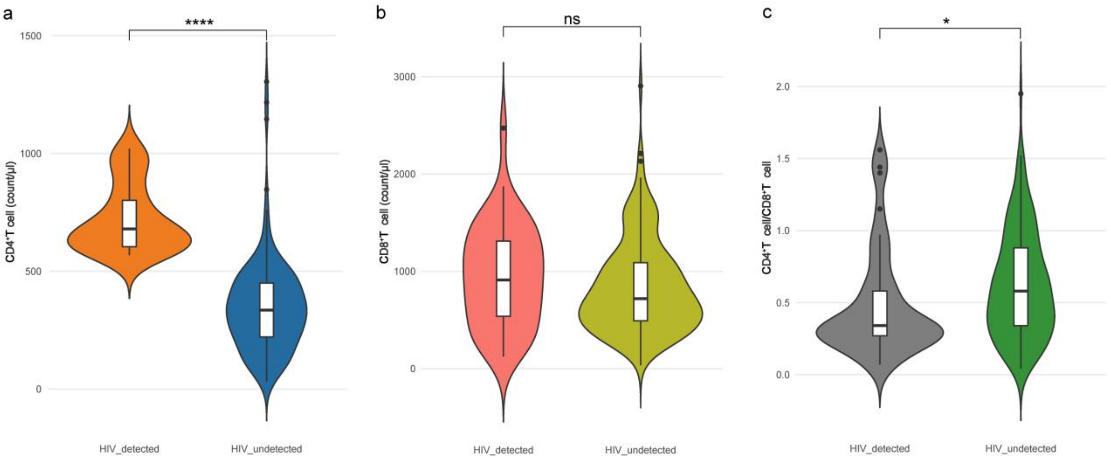

Comparison of CD4+ T-cell counts, CD8+ T-cell counts, and CD4+/CD8+ ratios between individuals with detectable HIV (HIV_detected, n=41, biologically independent samples) and individuals with undetectable HIV (HIV_undetected, n=163, biologically independent samples). Each violin plot illustrates the distribution of the data, with the box plot representing the interquartile range (IQR), and the median indicated by the line within the box (Welch's two-sample t-test). (a) A highly significant difference in CD4+ T-cell counts is observed between the HIV_detected group and the HIV_undetected group ($p<0.0001$，Cohen’s d= 2.0 [95% CI : 1.6, 2.4]). (b) CD8+ T-cell Count (cells/μL) showed no statistically significant difference (ns). (c) CD4+ T-cell /CD8+ T-cell Ratio showed significant differences between the HIV_detected group and the HIV_undetected group ($p=0.04$, Cohen's d = −0.37 [95% CI: −0.72, −0.03]).

Supplementary Figure 2. Comparison of viral composition across taxonomic levels.

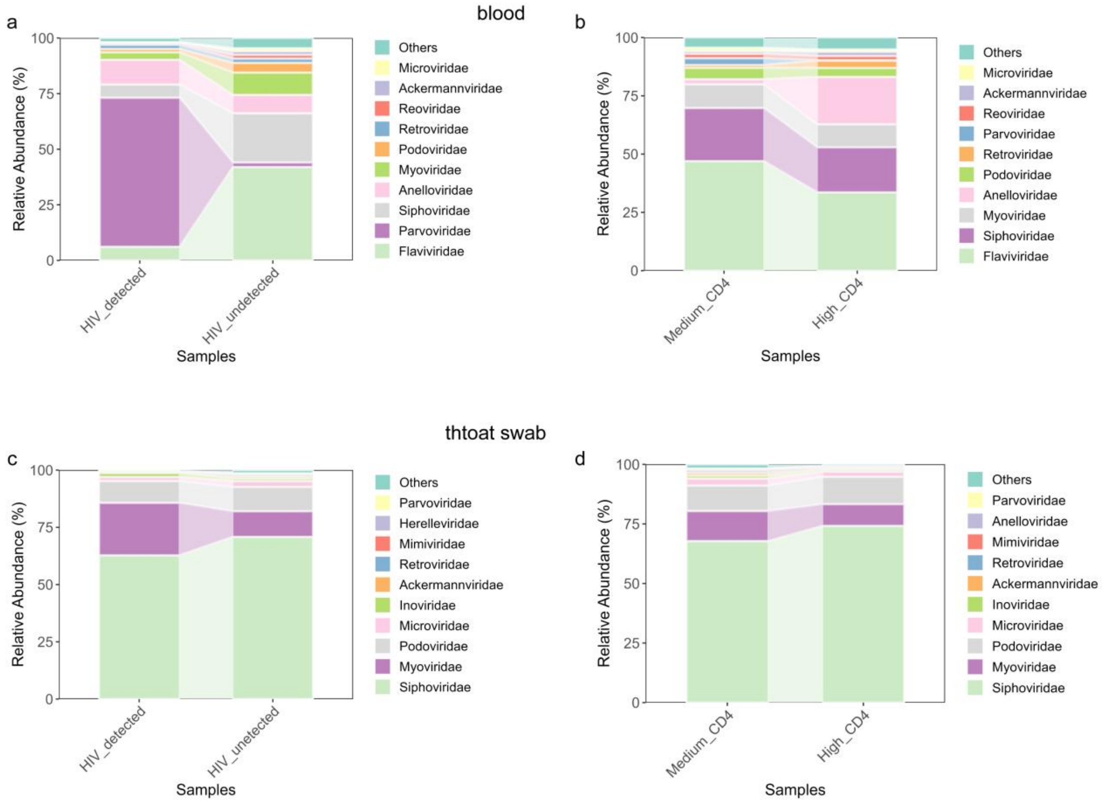

(a) and (b) show the relative abundance of the top 10 viral families in blood samples, illustrated by stacked bar charts. Specifically, panel (a) compares the HIV-detected and HIV-undetected paired groups, while panel (b) compares the Medium-CD4 and High-CD4 groups. Stacked bar charts in panels (c) and (d) illustrate the relative abundance of the top 10 viral families in throat swab samples, comparing the HIV-detected versus undetected groups and the Medium-CD4 versus High-CD4 groups, respectively.

Supplementary Figure 3. Analyses of viral diversities of the blood samples across taxonomic levels.

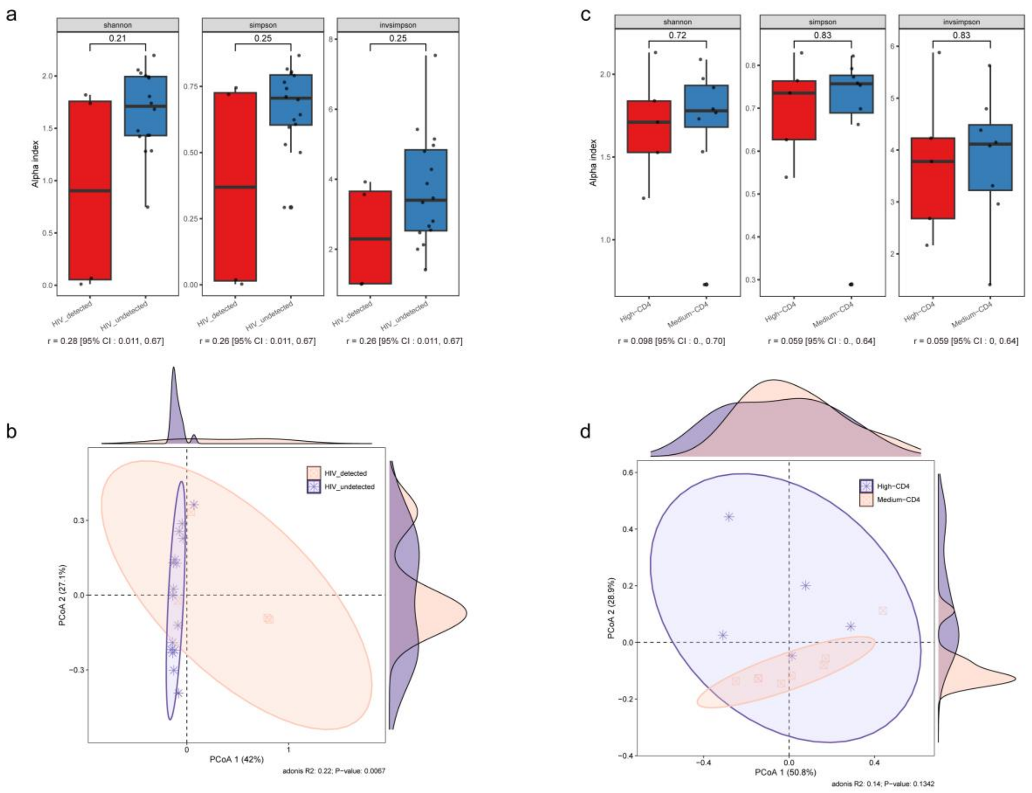

Panels (a) and (c) compare the alpha diversities at the family level between the HIV-detected (n=4, pools) and HIV-undetected (n=16, pools) groups, and between the Medium-CD4 (n=8, pools) and High-CD4 (n=5, pools) groups, respectively. Panels (b) and (d) present comparisons of beta diversity at the family level for two group pairs: HIV-detected vs. undetected, and Medium-CD4 vs. High-CD4, respectively.

Supplementary Figure 4. Analyses of viral diversities of the trhoat swab samples between Medium-CD4 and High-CD4 groups.

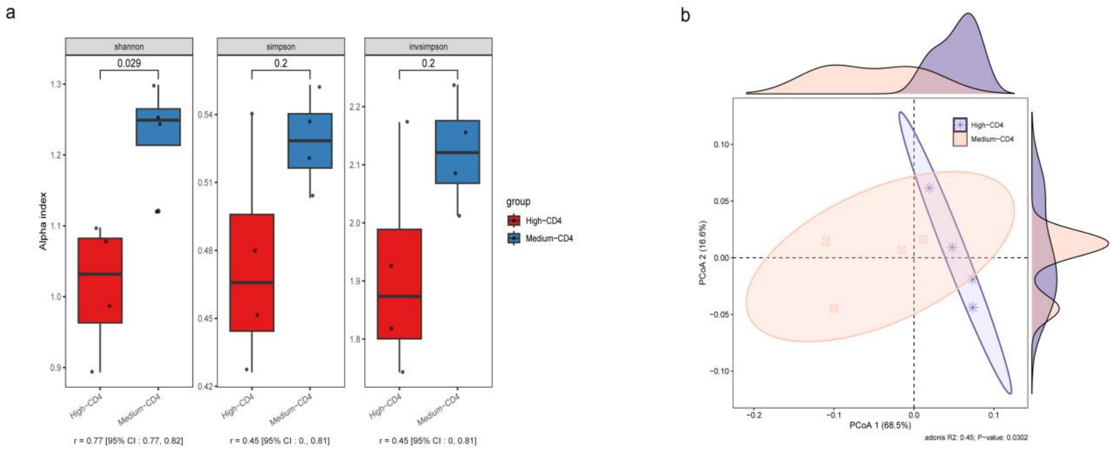

(a) The alpha diversities at the family levels was compared between Medium-CD4 (n=4, pools) and High-CD4 (n=4, pools) groups, using the Shannon, Simpson, and Inverse Simpson indices, respectively. (b) Beta diversities at the family levels was assessed.

Supplementary Figure 5. Differential Analysis with STAMP.

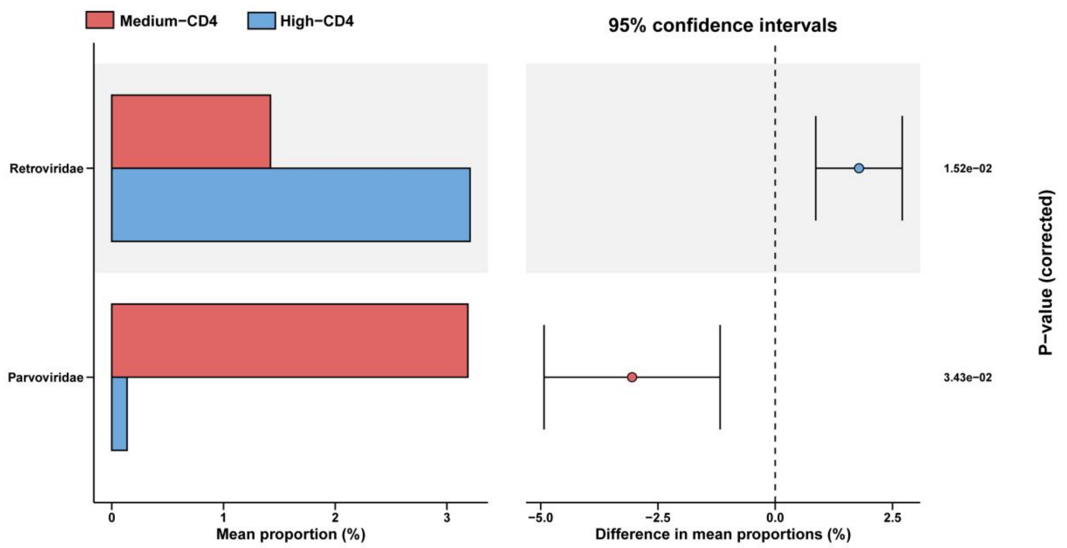

Differential analysis of the virome at the family level in blood samples between Medium-CD4 (n=8, pools) and High-CD4 (n=5, pools) groups. The bar charts in each individual figure show the relative abundance of specific species across different groups.

Supplementary Figure 6. Evolutionary Analysis and Viral Structure.

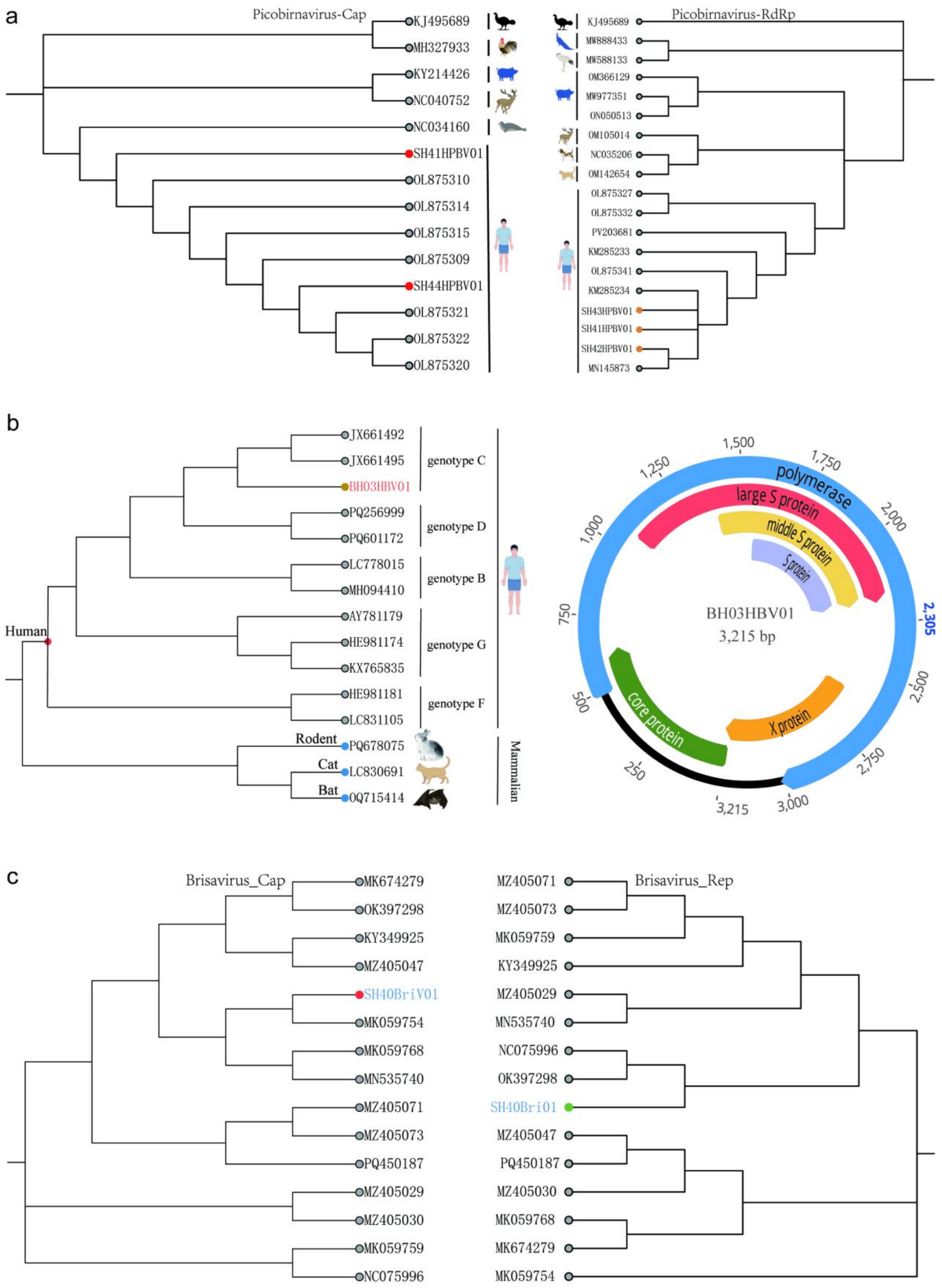

(a) Evolutionary analysis was conducted based on the Cap and RdRp genes of Picobirnavirus, with red and orange circles highlighting the viral sequences obtained in this study. (b) A phylogenetic tree was constructed based on the polymerase gene of HBV, with the viral sequences obtained in this study marked in red. On the right is the

full genome structure and functional protein annotation of HBV obtained in this study. (c) Evolutionary analysis was performed based on the Cap and Rep genes of Brisavirus, with red and green circles indicating the viral sequences obtained in this study. The viral genomic structure was produced with Geneious Primer V2025.0.2 software; the human and animal icons in the phylogenetic trees are from Figdraw under license (ID:ROPUAc4998).

Supplementary Figure 7. Gene Function Analysis. (a) Volcano plot of differentially expressed genes.

HIV_undetcted VS HIV_detcted

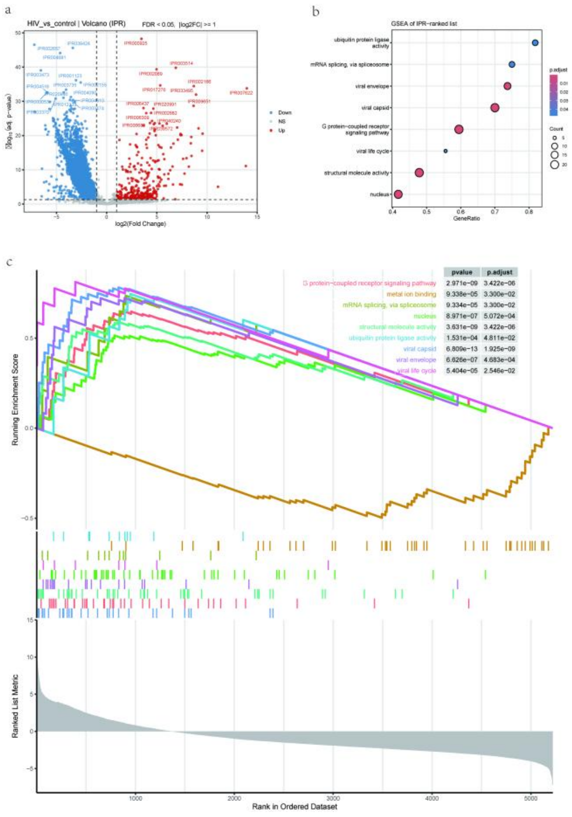

The x-axis represents the log2-transformed fold change in gene expression (log2(Fold Change)), and the y-axis shows the negative logarithm of the p-value (-log10(p-value)). Red and blue dots indicate significantly up-regulated and down-regulated genes in the HIV patient group, respectively (filtering criteria: $|log2FC| \geq 1$ and adjusted p-value (p.adjust) < 0.05). (b) GSEA enrichment results plot.The most significantly enriched (up-regulated) InterPro entries are displayed in ascending order of adjusted p-value (p.adjust). Different colors represent distinct InterPro entries. (c) GSEA enrichment profiles for up and down regulated GO

functional terms. The colored curves (red or blue) depict the running enrichment score (RES) as the analysis progresses through the ranked gene list (based on association strength with the phenotype). The peak of each curve indicates the point of maximal enrichment. Vertical tick marks represent the positions of the core enriched InterPro entries within the ranked list. The gray bars at the bottom of each plot illustrate the distribution of all genes associated with InterPro entries across the ranked list.

Supplementary Figure 8. Gating strategy and cell subset quantification.

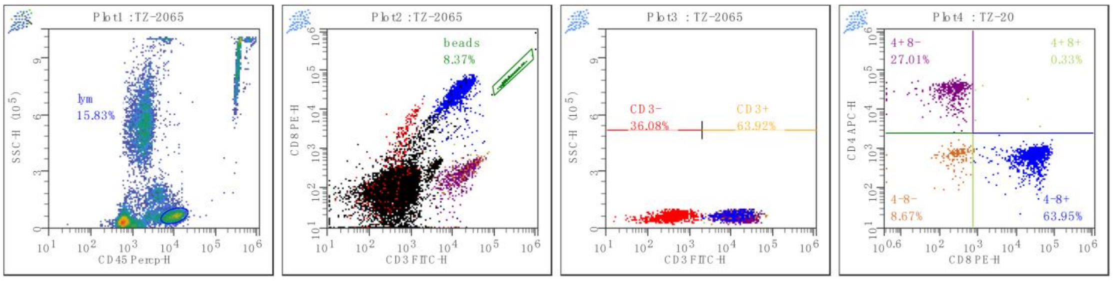

TZ-2065	Events	% Total	% Parent	% GrandParent	Absolute Count
lym	2375	15.83%	15.83%	15.83%	1823
CD3-	857	5.71%	36.08%	5.71%	658
CD3+	1518	10.12%	63.92%	10.12%	1165
4+8-	410	2.73%	27.01%	17.26%	315
4+8+	5	0.03%	0.33%	0.21%	4
4-8-	137	0.87%	8.67%	5.55%	101
4-8+	966	6.46%	63.95%	40.89%	745
beads	1256	8.37%	8.37%	8.37%	—

Representative flow cytometry gating hierarchy and cell counts from one patient sample. The table shows the number of events, percentage of total events, percentage of parent gate, percentage of grandparent gate, and absolute count (cells/ μ L) for each indicated lymphocyte subset.The gating sequence was as follows: **(1)** Leukocytes were first identified as $CD45^{+}$ cells on a CD45 PerCP-H vs. SSC-H plot, and the lymphocyte population (lym) was subsequently gated. **(2)** $CD3^{-}$ and $CD3^{+}$ T-cell populations were distinguished from lymphocytes using CD3 FITC-H. **(3)** Among $CD3^{+}$ T cells, the $CD4^{+}$ $CD8^{-}$, $CD4^{+}$ $CD8^{+}$, $CD4^{-}$ $CD8^{-}$, and $CD4^{-}$ $CD8^{+}$ subsets were further resolved on a CD4 APC- H vs. CD8 PE- H plot. **(4)** Beads were used for absolute count calibration (the bead population is shown in the CD45 vs. SSC plot). This gating strategy was uniformly applied to all 203 patient

samples.
