# Supplementary material for: HIV-driven virome dysbiosis unveils distinct virome features and inter-viral correlations in blood and respiratory niches
Source: Commun Biol. 2026 May 8;9:958. doi: 10.1038/s42003-026-10221-z (PMC13369948; doi:10.1038/s42003-026-10221-z)
Supplement: Supplementary file 2 — Description of Additional Supplementary Materials [file 42003_2026_10221_MOESM2_ESM.pdf]

Description of Additional Supplementary Files

File name: Supplementary Data 1

Description: Immune Cell Levels, HIV Viral Load, and ART Duration

File name: Supplementary Data 2

Description: Quantification of CMV and EBV viral loads

File name: Supplementary Data 3

Description: Sequencing quality statistics

File name: Supplementary Data 4

Description: Viral read counts at the taxonomic family level

File name: Supplementary Data 5

Description: Viral read counts at the taxonomic genus level

File name: Supplementary Data 6

Description: Viral read counts at the taxonomic species level

File name: Supplementary Data 7

Description: Viral read counts in blood samples, stratified by taxonomic level, for LEfSe (LDA Effect Size) analysis

File name: Supplementary Data 8

Description: Viral read counts in throat swab samples, stratified by taxonomic level, for LEfSe (LDA Effect Size) analysis

File name: Supplementary Data 9

Description: Input counts for InterPro-based functional analysis (InterPro2GO; DIAMOND+MEGAN)
